# Leveraging Electrochemical Diversity in Engineering Liquid‐State Ionic Devices for Neuromorphic Computing

**DOI:** 10.1002/smll.202511663

**Published:** 2026-01-21

**Authors:** Yechan Noh, Alex Smolyanitsky

**Affiliations:** ^1^ Applied Chemicals and Materials Division National Institute of Standards and Technology Boulder USA; ^2^ Department of Physics University of Colorado Boulder Boulder USA; ^3^ Department of Aerospace and Mechanical Engineering University of Notre Dame Notre Dame USA; ^4^ Present address: National Institute of Standards and Technology and University of Notre Dame Notre Dame USA

**Keywords:** 2D membranes, ion transport, molecular dynamics, nanopore, neuromorphic computing

## Abstract

Implementing the brain's electrochemical principles in liquid‐state ionic devices for neuromorphic computing has gained notable momentum. A unique advantage of such devices is the abundance of ions and molecular species available for electrochemical signaling. In this work, we demonstrate that electrochemical diversity translates into functional diversity within the barrier‐limited transport regime, a phenomenon not captured by classical diffusion transport. Using molecular dynamics simulations, we investigate multi‐ionic transport through Ångström‐scale pores and reveal diverse behaviors, including voltage‐inactivated transport, electrochemical pulse generation, and synaptic potentiation. The resulting design space scales exponentially with the number of ion species, as $2^{N_{\text{ion}}}$, yielding an astronomically large number given the existence of more than 100 known ionic species. Our work highlights an extensive, unexplored design space of electrochemical computing devices occurring in the barrier‐limited transport regime.

## Graphical abstract

Mixed‐ion transport through Å‐scale pores exhibits various functions, including voltage activation, voltage inactivation, nanopulse generation, and synaptic behavior. Our work reveals that one of the fundamental advantages of ionic computing over electronic computingthe diversity of charge carriersdirectly translates into functional diversity in the barrier‐limited regime.

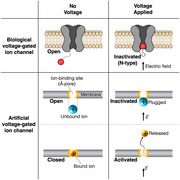

## 1 Introduction

Biological neural networks achieve energy‐efficient learning and sensory processing by precisely regulating the transport of ions and molecules through electrically, chemically, and mechanically gated channels operating in an aqueous environment [1, 2, 3, 4]. In contrast, today's artificial intelligence (AI) systems rely on electrons as signal carriers in solid‐state devices, while the actual neural network functionality is emulated in software. Fueled by the ever‐increasing energy cost of using such neural networks [5], there is growing interest in directly replicating the brain's molecular‐level mechanisms to enable highly efficient AI. Recent advances in nanofluidics and nanofabrication have positioned liquid‐state ionic devices as a promising platform for mimicking nature's molecular‐scale functions, such as ion gating and single‐species selectivity. Beyond biomimetics, liquid‐state ionics has shown promise in a range of engineering applications including rechargeable batteries [6], which have already demonstrated significant practical viability, as well as emerging areas such as separation processes [7, 8], osmotic energy harvesting [9, 10], neuromorphic computing [11, 12, 13, 14], mechanotransduction [15, 16], and DNA sequencing [17, 18].

One of the key requirements for biomimetic liquid‐state ionics is achieving barrier‐limited transport, where the transport process is governed by ion‐specific energy landscapes, which is fundamentally distinct from the diffusive transport paradigm [19]. The conventional diffusive transport theories are characterized by a diffusion coefficient and the potential gradient:

(1) I∝−D∇ψ,

where D is the diffusion coefficient and ∇ψ denotes the gradient of the relevant potential (e.g., chemical or electrical potential). In contrast to diffusive transport, barrier‐limited transport is exponentially sensitive to the local free energy barriers encountered along the conduction pathway, enabling highly selective atomic transport. When a single energy barrier dominates, the overall ionic current, I, can be modeled by an Arrhenius‐type equation [20], as described by transition state theory:

(2) $$I\propto \exp\left(-\frac{\Delta E}{k_{B}T}\right),$$

where ΔE is the rate‐setting energy barrier, $k_{B}$ is the Boltzmann constant, and T is the temperature. Such barrier‐limited transport underlies essential biological functions, such as the single‐ion selectivity of certain biological potassium channels [21, 22] and the highly selective water transport through aquaporin‐1 channels [23, 24]. In synthetic membranes, barrier‐limited transport has been explored in various contexts, including mechanosensitive ion conduction [16, 20], high cation‐cation selectivity [8, 25], and water desalination [7, 8]. Importantly, the competitive transport and trapping of different types of ions create memristive and memcapacitive behavior [11, 12], exhibiting GHz‐scale conductance switching and exceptionally low energy costs of 0.1–100 aJ per voltage spike [12].

**FIGURE 1:**
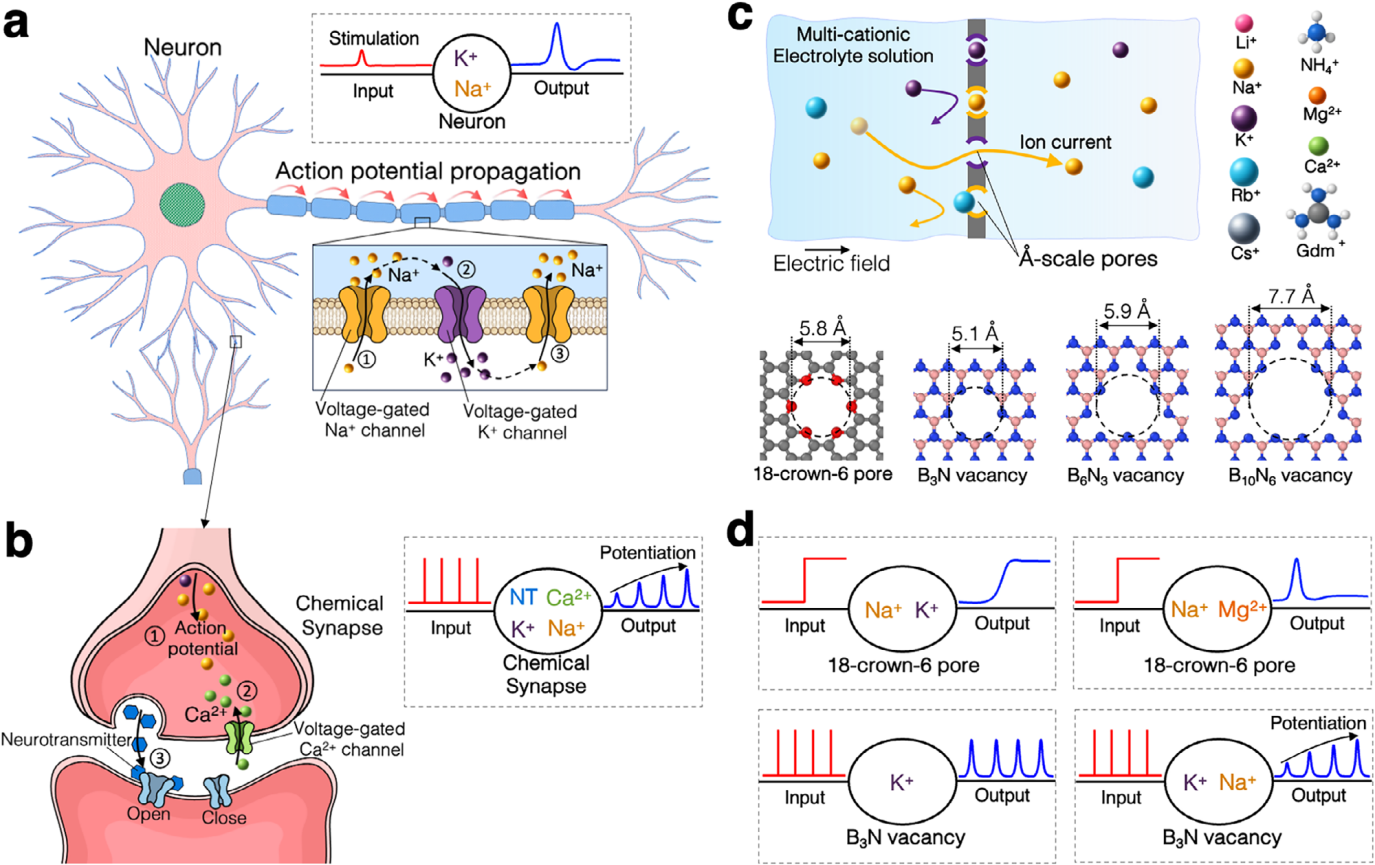
Multi‐salt processes in biological and synthetic systems. (a) Illustration of action potential propagation in a neuron, mediated by voltage‐gated ${\mathrm{Na}}^{+}$ influx and $K^{+}$ efflux. The inset (gray dashed box) shows a simplified input–output diagram of the postsynaptic action potential in response to an electrical stimulus at the presynaptic site, along with the associated ions and system. (b) Potentiation at a chemical synapse, illustrating voltage‐gated ${\mathrm{Ca}}^{2+}$ entry, neurotransmitter release, and postsynaptic potentiation. The inset shows an input–output diagram of postsynaptic potentiation in response to a train of presynaptic action potentials. (c) Schematic of the system used in all‐atom molecular dynamics simulations of multi‐cation transport through Å‐pores under an applied electric field in a mixed‐cation electrolyte. Bottom: atomic structures and effective diameters of four representative Å‐pores. (d) Input (voltage)–output (ion current) diagrams of representative synthetic multi‐ionic systems: voltage activation (top left), voltage inactivation (top right), non‐potentiation (bottom left), and potentiation (bottom right).

Biological systems exploit ionic diversity in more sophisticated ways—utilizing approximately 20 different ion types along with various biomolecules [26]. As illustrated in Figure 1a, neurons generate action potentials through the sequential activation of voltage‐gated ${\mathrm{Na}}^{+}$ and $K^{+}$ channels [27]. A well‐characterized example of synaptic plasticity in chemical synapses also involves the coordinated action of multiple species—including ${\mathrm{Na}}^{+}$, $K^{+}$, ${\mathrm{Ca}}^{2+}$, and neurotransmitter molecules (Figure 1b) [28]. Despite this rich variety of ionic and molecular species that support complex biological functions, liquid‐state ionics with barrier‐limited transport have only begun to explore this richness—limited so far to just a few example cases studied very recently. Expanding this line of research could unlock a broad range of yet‐unrealized device functionalities.

In this work, we take an initial step toward exploring ionic behavior arising from electrochemical diversity within the barrier‐limited transport regime. Using extensive all‐atom molecular dynamics simulations, we investigate nine cation species across four distinct Å‐pores, yielding a combinatorial design space of over 2000 unique ionic mixture–pore structure combinations. By directly examining 24 representative cases involving two distinct cation types, we uncover subsets of a massive design space enabled by electrochemical diversity. These findings highlight a potentially compelling research direction for iontronics and the nanofluidics community, wherein ion‐based logic devices can exploit their inherent electrochemical diversity to achieve levels of functional richness and nonlinearity that exceed those attainable with electron‐based logical elements. Finally, we introduce a functional classification of cations for each Å‐scale pore, based on their roles in the targeted design of liquid‐state ionic devices.

## 2 Results and Discussion

To explore the impact of electrochemical diversity on ion transport dynamics, we constructed model systems based on two‐dimensional membranes featuring electrically neutral Å‐scale pores immersed in aqueous salt solutions. These pores (Figure 1c) present well‐defined, barrier‐limited pathways that selectively permit cation transport while remaining virtually impermeable to anions, making them ideal for studying competitive ion transport and selective binding phenomena. We selected nine chemically diverse cations—including monovalent, divalent, and multiatomic species—to span a wide range of charge, size, and hydration characteristics. Figure 1d presents simplified input–output diagrams of the key transport behaviors observed in our simulations, including voltage activation, inactivation, nanopulse generation, and synaptic potentiation, each of which will be discussed in detail in the following sections.

### 2.1 Ion Transport Characteristics of Single‐Salt Systems

**FIGURE 2:**
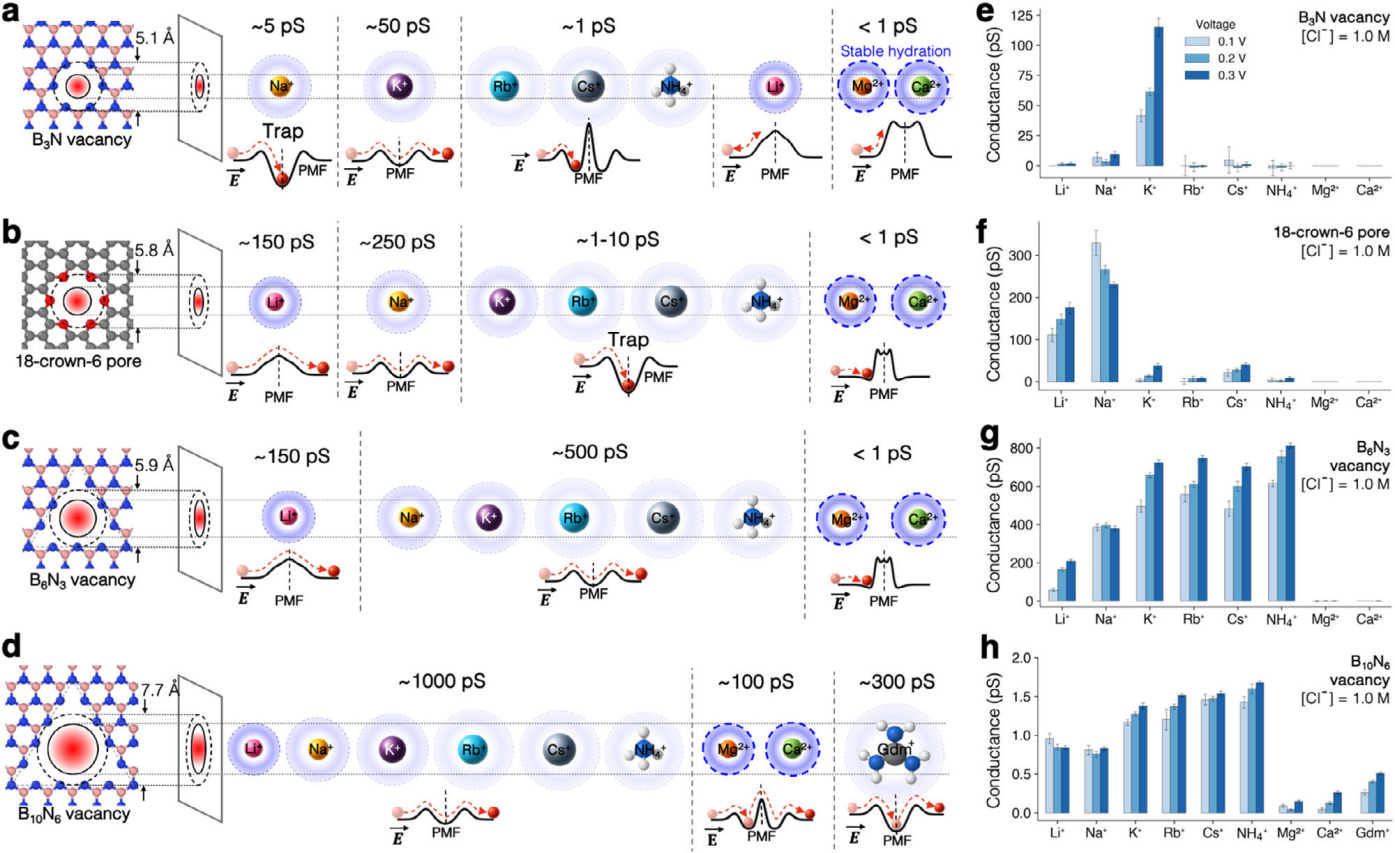
Transport behavior of various cations in single‐salt systems across four distinct Å‐pores. Transport characteristics of various cations are shown for (a) $B_{3}N$ vacancy, (b) 18‐crown‐6 pore, (c) $B_{6}$ $N_{3}$ vacancy, and (d) $B_{10}$ $N_{6}$ vacancy. The pore diameter is evaluated by the largest inscribed circle within the pore based on the center position of the edge atoms. Ion transport schematic illustrates the transport/trapping mechanism under an electric field. Dashed lines in the PMF indicates the pore center. Single‐pore conductance values are summarized in (e–h) for (e) $B_{3}N$ vacancy, (f) 18‐crown‐6 pore, (g) $B_{6}$ $N_{3}$ vacancy, and (h) $B_{10}$ $N_{6}$ vacancy under cross‐membrane voltage bias of 0.1 V, 0.2 V, and 0.3 V. All systems were simulated in 1.0 M ${\mathrm{Cl}}^{-}$ electrolyte, with monovalent cations at 1.0 M and divalent cations at 0.5 M concentrations.

Before investigating ion transport involving multiple salts, we first examined single‐salt systems. Figure 2a–d shows the diverse, ion‐specific transport behaviors observed for each considered ion–pore combination. As shown in Figure 2a,b, the $B_{3}N$ vacancies in hBN exhibit highly selective $K^{+}$ transport, while the 18‐crown‐6 pores in graphene are selective for ${\mathrm{Li}}^{+}$ and ${\mathrm{Na}}^{+}$. Note that the size of the $B_{3}N$ vacancy is 0.7 Å smaller than the 18‐crown‐6 pore; however, it conducts the larger $K^{+}$ ion more efficiently than the smaller ${\mathrm{Li}}^{+}$ and ${\mathrm{Na}}^{+}$ ions (Figure 2a, b). This example underscores the complexity of barrier‐limited transport, which goes beyond the intuitive pore size‐dependent steric exclusion based on ionic radii and pore size [16]. Instead, ion transport is governed by complex barrier formation mechanisms, including ion solvation energetics [29], ion–pore coordination chemistry [8], and entropic barriers [15, 30]. Find more detailed discussion on the barrier formation in the section S2 in supporting information.

In addition to transport, ion trapping is a key feature in barrier‐limited transport that regulates the conductive state of each pore. 18‐crown‐6 ether is known for effectively traps $K^{+}$, ${\mathrm{Rb}}^{+}$, ${\mathrm{Cs}}^{+}$, and ${\mathrm{NH}}_{4}^{+}$ ions to its coordination geometry [31]. Similarly, the 18‐crown‐6 pore embedded in graphene traps all these ions, including $K^{+}$ and ${\mathrm{Rb}}^{+}$, as reported in earlier work [20] (Figure 2b). The relatively smaller $B_{3}N$ vacancy pore selectively traps ${\mathrm{Na}}^{+}$ through optimal coordination with six nitrogen atoms at the pore edge. The larger 16‐atom vacancy pore ($B_{10}$ $N_{6}$ vacancy) is capable of trapping multi‐atomic ions such as, ${\mathrm{Gdm}}^{+}$ (Figure 2d), due to its favorable match of shape, size, and binding affinity. Notably, the application of an electric field across the membrane causes certain ions to become trapped at the pore entrance. For example, the $B_{3}N$ vacancy in hBN is nearly impermeable to ${\mathrm{Rb}}^{+}$, ${\mathrm{Cs}}^{+}$, and ${\mathrm{NH}}_{4}^{+}$ ions due to their large ionic radii. However, under an external electric field, these ions are pushed against the repulsive barrier and become trapped at the pore entrance.

The free energy landscape is a key factor governing both ion transport and trapping in single‐salt systems. While a wide variety of PMF profiles exist (see Figure S2, Supporting Information), we highlight some representative shapes shown in the insets of Figure 2a–d, along with schematics depicting ionic behavior under a moderate electric field (see Supporting Information Figure S2 for the full PMF dataset for each cation–pore pair). The $B_{3}N$ vacancy exhibits five representative PMF shapes (Figure 2a): 1) ${\mathrm{Na}}^{+}$ encounters a moderate dehydration barrier at the pore aperture followed by a deep energy minimum at the center, leading to trapping; 2) $K^{+}$ exhibits a shallow energy minimum at the pore center due to a repulsive potential arising from a slight size mismatch, which facilitates permeation [16]; 3) Larger monovalent cations (${\mathrm{Rb}}^{+}$, ${\mathrm{Cs}}^{+}$, and ${\mathrm{NH}}_{4}^{+}$) exhibit a pronounced steric peak at the pore center, along with a local PMF minima on either side of the pore; 4) ${\mathrm{Li}}^{+}$, despite its small ionic radius, retains a tightly bound hydration shell that creates a high dehydration barrier, resulting in minimal conduction; 5) Divalent ions, such as ${\mathrm{Mg}}^{2+}$ and ${\mathrm{Ca}}^{2+}$, encounter steep dehydration barriers at the pore entrance due to entropic barrier [30].

### 2.2 Voltage Gating Through Ion Transport Competing for Pore Sites in Å‐pore Arrays

**FIGURE 3:**
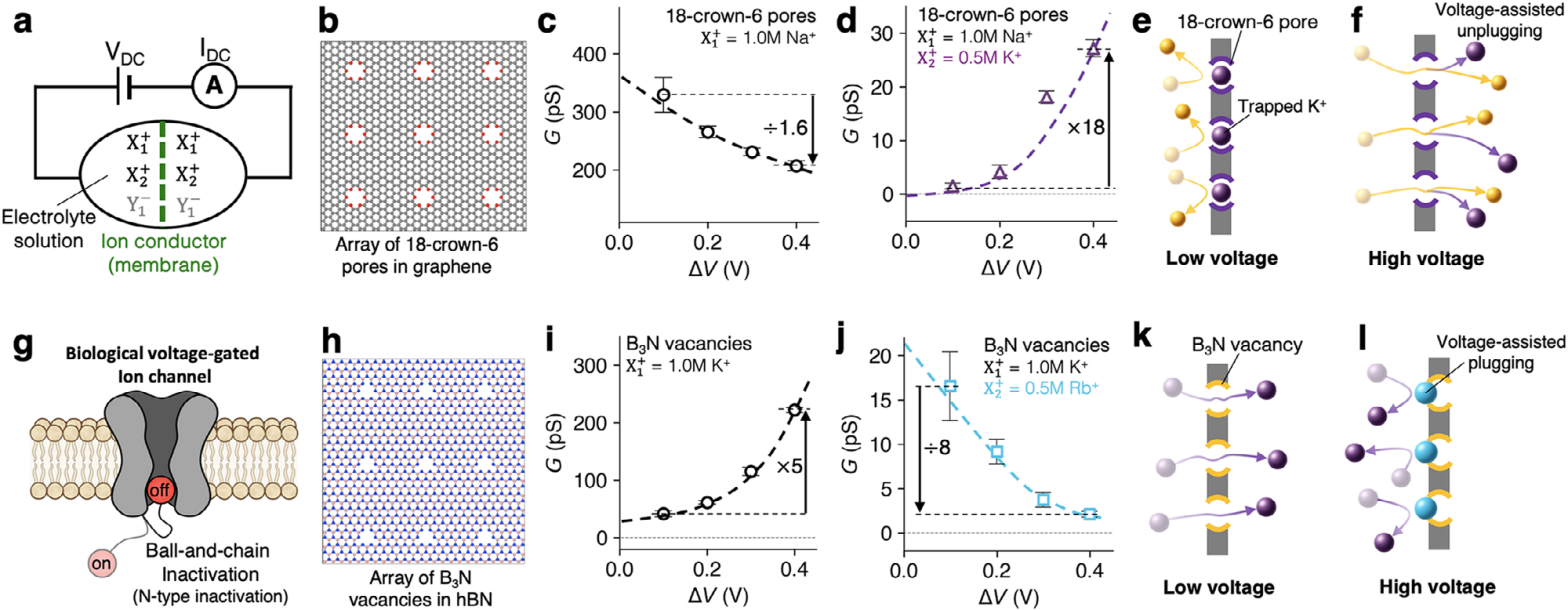
Voltage activation and inactivation in binary salt systems. (a) Schematic of ion conduction through an Å‐pore array in a 2D membrane under a DC voltage bias ($V_{\mathrm{DC}}$), involving the transport of two cation species ($X_{1}^{+}$ and $X_{2}^{+}$) and one anion type ($Y_{1}^{-}$=${\mathrm{Cl}}^{-}$) in solution. (b) Array of 18‐crown‐6 pores embedded in graphene. (c,d) Conductance as a function of applied voltage for 18‐crown‐6 pore arrays with (c) 1.0 M NaCl and (d) mixed 1.0 M NaCl / 0.5 M KCl. (e,f) Schematics of the voltage‐activation mechanism: (e) at low voltage, the pore is blocked by $K^{+}$; (f) at high voltage, $K^{+}$ is removed via voltage‐assisted unplugging. (g) Illustration of a biological voltage‐gated ion channel employing N‐type (ball‐and‐chain) inactivation. (h) Array of $B_{3}N$ vacancies in hBN. (i,j) Conductance–voltage plots for $B_{3}N$ vacancy arrays with (i) 1.0 M KCl and (j) mixed 1.0 M KCl / 0.5 M RbCl. (k,l) Schematics of voltage inactivation via voltage‐assisted ion plugging: (k) at low voltage, the pores are open; (l) at high voltage, ${\mathrm{Rb}}^{+}$ ions block the pores.

Building on our understanding of cation transport in single‐salt systems, we investigate ion transport involving two different cations (see the system schematics in Figure 3a). In the 18‐crown‐6 pore array (Figure 3b) with a single‐salt 1.0 M NaCl solution, ${\mathrm{Na}}^{+}$ ions conduct efficiently, exhibiting a low‐voltage (ΔV≈0.1 V) conductance of approximately 350 pS per pore (Figure 3c). However, upon the addition of 0.5 M KCl to this system, the low‐voltage conductance drops by over two orders of magnitude to approximately 1 pS (Figure 3d) and exhibits a highly nonlinear conductance response to mixed salt conditions: G(1.0 M NaCl+0.5 M KCl)≪G(1.0 M NaCl)+G(0.5 M KCl) (see Figures 3c,d and 2f). As the transmembrane bias increases, the conductance of the mixed system recovers exponentially, rising by more than an order of magnitude by 0.3 V. This behavior is driven by voltage‐assisted unplugging of $K^{+}$ ions favorably trapped within the crown cavities (Figure 3e,f) and thus $K^{+}$ ions act as pore blockers at low voltage that can be expelled under higher bias, restoring ${\mathrm{Na}}^{+}$ conduction. This voltage‐gated ${\mathrm{Na}}^{+}$ transport mimics the function of biological voltage‐gated ${\mathrm{Na}}^{+}$ channels [32], but achieves the gating without requiring conformational changes in the pore.

In contrast to the voltage‐activation, the $K^{+}$‐${\mathrm{Rb}}^{+}$ cation pair in the case of $B_{3}N$ vacancy array (Figure 3h) exhibits voltage‐inactivation, analogous to N‐type (ball‐and‐chain) inactivation of biological voltage‐gated channels (Figure 3g), where a protein segment, known as the “ball” at the pore mouth blocks the pore lumen to halt ion flow [33]. In our system, however, free ions in solution act as the “balls,” achieving inactivation without structural appendages of pores themselves. In a single‐salt, 1.0 M KCl, the membrane exhibits $K^{+}$ conductance increasing as a function of voltage, rising from ∼40 pS at 0.1 V to ∼200 pS at 0.4 V (Figure 3i). Upon the addition of 0.5 M RbCl, this trend is dramatically reversed to voltage inactivation (Figure 3j). The conductance drops from ∼16 pS at 0.1 V to ∼2 pS at 0.4 V, decreasing by a factor of eight. This results in pronounced nonlinearity with increasing voltage: G(1.0 M KCl+0.5 M RbCl)≪G(1.0 M KCl)+G(0.5 M RbCl) (see Figures 3i,j and 2e). This suppression of ion transport at higher voltages arises from voltage‐assisted plugging of ${\mathrm{Rb}}^{+}$ ions at the entrance of $B_{3}N$ vacancies (Figure 3k–l). In this process, ${\mathrm{Rb}}^{+}$ is pushed by the external field against the repulsive ion‐pore barrier. As a result, ${\mathrm{Rb}}^{+}$ ions become trapped near the pore entrance, blocking $K^{+}$ transport under high‐voltage conditions (e.g., ∼0.4 V).

Although this study focuses on bias voltage ranges near the biological regime (∼100 mV), it is instructive to consider the asymptotic high‐voltage limits of competitive ion transport. For voltage‐activated systems, sufficiently large bias would drive the effective synaptic weight toward unity, which leads to a disappearance of any transport competition; in this limit, for a binary mixture of constituents A and B, the total conductance approaches a linear superposition G(A+B)=G(A)+G(B). Conversely, for voltage‐inactivated systems at high biases, the weight asymptotically approaches zero, corresponding to complete suppression of ion flow, G(A+B)=0. These asymptotic trends define the natural saturation limits for each functional regime.

It is also worth mentioning that pore spacing can, in principle, influence ion transport behavior when the distance between pores becomes comparable to or smaller than the Debye length. In our study, we focused on the high‐concentration regime (1.0–1.5 M), where the center‐to‐center pore spacing in our membranes (1.5–1.7 nm) is much larger than the Debye length (∼0.3 nm). Therefore, the voltage gating observed here is not sensitive to pore spacing, as confirmed by the case study using different pore spacings shown in Figure S7 (Supporting Information).

### 2.3 Voltage Gating Dynamics and Memory Effects

**FIGURE 4:**
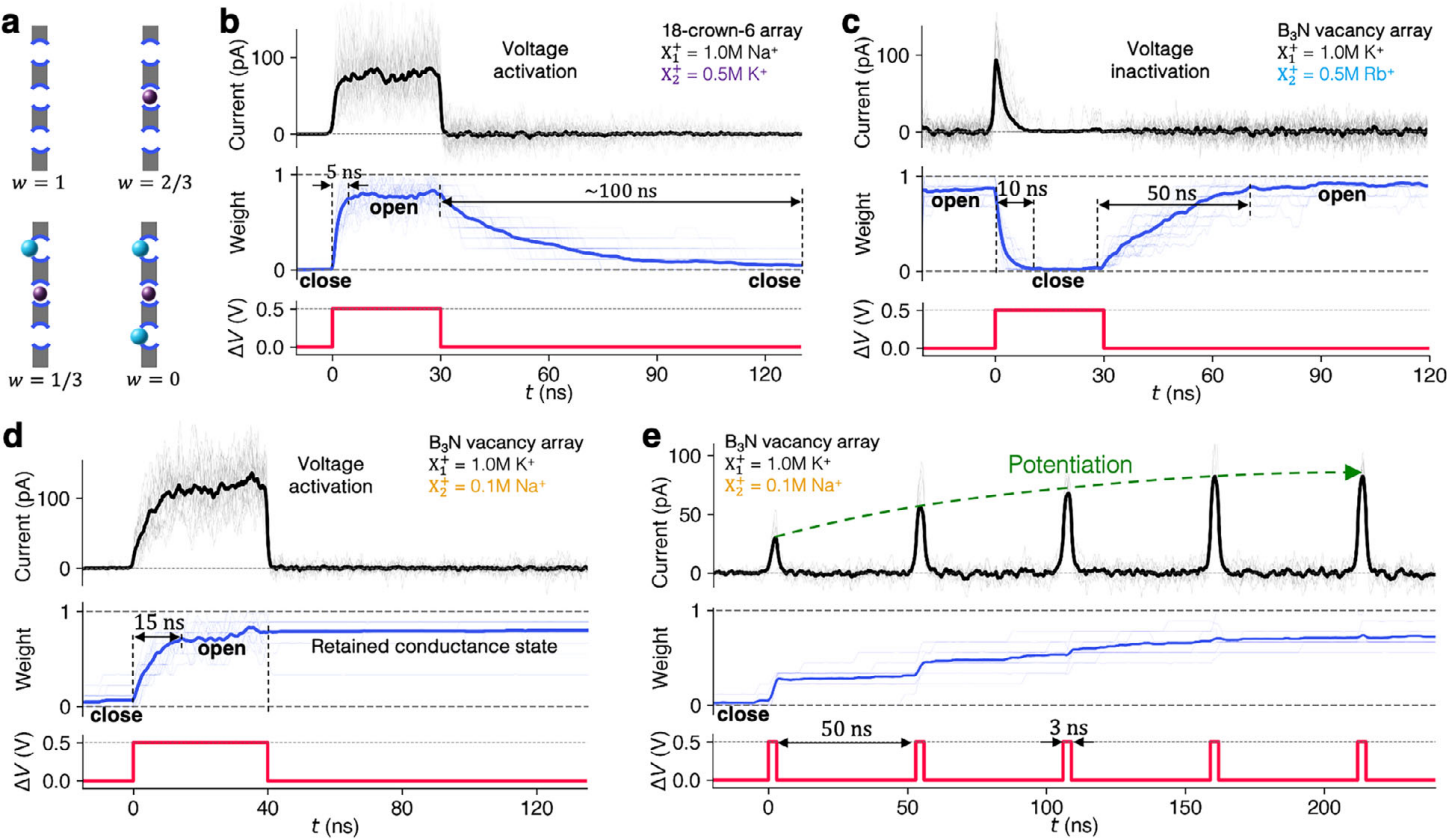
Dynamics of voltage‐activation and inactivation. (a) Definition of conductance weight, w. (b–e) Time‐resolved simulations showing transient current (top), conductance weight w (middle), and applied voltage (bottom): (b) Off–on–off cycle of voltage activation in 18‐crown‐6 pore array within a 1.0 M NaCl and 0.5 M KCl solution, averaged over 40 independent simulations; (c) on–off–on cycle of voltage inactivation in $B_{3}N$ vacancy array in a 1.0 M KCl and 0.5 M RbCl solution, averaged over independent 20 simulations. (d) Off–on‐off cycle of voltage activation in $B_{3}N$ in a 1.0 M KCl and 0.5 M NaCl solution, averaged over 20 independent simulations; (e) Dynamic conductance switching by voltage pulses in $B_{3}N$ in a 1.0 M KCl and 0.5 M NaCl solution, averaged over 10 independent simulations. Thick lines represent the averaged data; thin lines show individual traces.

To investigate gating dynamics, we quantify the conductance state of an Å‐pore membrane using the conductance weight [11, 12], w, defined as the ratio of unblocked (i.e., open) pores to the total number of pores in the membrane (Figure 4a). The overall membrane conductance is then expressed as a simple linear weighted function [11, 12]:

(3) $$G=wG_{0},$$

where $G_{0}$ denotes the conductance of the membrane when all pores are fully open. To investigate the transient dynamics and reversibility of ionic gating, we applied a step voltage input in which the bias was abruptly raised to 0.5 V and then returned to zero.

Shown in Figures 4b–d are the dynamic responses of ion current and weight during the voltage activation and inactivation. For the ${\mathrm{Na}}^{+}$‐$K^{+}$ pair and the 18‐crown‐6 pore array in graphene (Figure 4b), we observe rapid voltage activation: within 5 ns of applying a high transmembrane bias (0.5 V), the conductance weight increases sharply from 0 to ∼0.8, indicating a transition from a ‘closed’ to an “open” state as $K^{+}$ ions are rapidly expelled from the pores. Upon removing the voltage bias, the conductance gradually decays back to the closed state over 100 ns as $K^{+}$ is reabsorbed by the crown pores. Conversely, the $B_{3}N$ vacancy array with the $K^{+}$‐${\mathrm{Rb}}^{+}$ pair exhibits voltage inactivation (Figure 4c): upon bias application, the system transitions from an open to a closed state within 10 ns, resulting in a current pulse of duration determined by how long it takes for the pores to be plugged. Once the bias is removed, the conductance weight gradually increases back to ∼0.9 over 50 ns as ${\mathrm{Rb}}^{+}$ ions are released into the surrounding solution. Notably, these ionic gating transitions occur on nanosecond timescales—several orders of magnitude faster than the micro‐to‐millisecond‐scale operations of their biological counterparts [34, 35]. Note that the pore's conductance state—whether ‘open’ or “closed,” as defined by the conductance weight—does not necessarily imply the presence or absence of ionic current. Current flows only when the pore is open and in the presence of driving forces, such as a voltage or chemical potential gradient, across the membrane.

During voltage‐activated transport, the memory retention time is governed by the characteristic timescale for re‐trapping the pore‐blocking ions. By selecting specific combinations of ion pairs, pore structures, and ionic concentrations, the retention dynamics can in fact be engineered. Figures 4b and 4d show two representative examples that exhibit distinct retention behaviors. Upon removal of the voltage bias, the system shown in Figure 4d retains the activated conductive state for a substantially longer duration than the system in Figure 4b, without requiring additional energy expenditure to maintain it. This characteristic of the $K^{+}$‐${\mathrm{Na}}^{+}$ pair in the case of $B_{3}N$ vacancies yields functionality of a 2D ionic memristor, as described in detail in our previous work [12] with a conductance state that is dynamically tunable by applied voltage pulses (see Figure 4e).

The estimated energy consumption of voltage‐gating in 2D membranes is remarkably low. The energy dissipated during conductance switching is given by [12]

(4) $$\Delta E=\int_{0}^{\tau }I\left(t\right)V\left(t\right)dt,$$

where I(t) and V(t) is the ion current and voltage bias, respectively, and integration is over the activation/inactivation time τ. This estimate offers a lower bound on energy cost—about 0.3 aJ for activation and 0.1 aJ for inactivation per pore.

There are earlier studies that demonstrated conductance control through blockage of larger atomic clusters [36, 37] and flexible polymeric chains [38]. In contrast, the gating mechanism presented here is enabled by a single ion, offering a fundamental advantage for high information density—especially when implemented in synthetic porous materials such as two‐dimensional metal–organic frameworks (2D MOFs), covalent organic frameworks (COFs), and other nanostructured architectures.

### 2.4 Design Space and Functional Mapping

**FIGURE 5:**
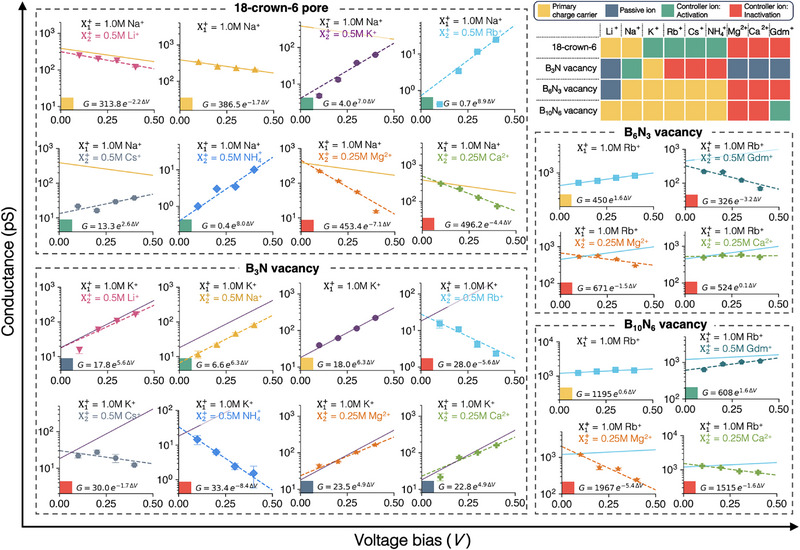
Voltage‐dependent conductance across diverse cation pairs in four Å‐pores. Each panel presents the log(G) versus ΔV relationship. The four pore types include the 18‐crown‐6 pore (top left), $B_{3}N$ vacancy (bottom left), $B_{6}$ $N_{3}$ vacancy (middle right), and $B_{10}$ $N_{6}$ vacancy (bottom right). Lines represent exponential fits of the form $G=Ce^{\alpha \Delta V}$ for single‐salt systems (solid line) and binary salt systems (dashed line), where the unit of alpha is $V^{-1}$. The colored square at the bottom left corner of each plot indicates the functional role of the secondary ion ($X_{2}^{+}$) or the conducting ion in the mono‐cationic system, as classified in the color map (top right).

As discussed above, mixing different ionic species in ionic systems can produce a wide range of functionalities in ionic devices, thereby exponentially expanding the design space with the number of ionic species available. The number of possible ionic mixtures selected from $N_{\text{ion}}$ ionic species is given by $2^{N_{\text{ion}}}-1$ for each distinct pore structure. For four different pores and nine cationic species, this yields $4(2^{9}-1)=2044$ combinations. When restricted to bicationic systems, the design space becomes Npores×Nion2=4×92=144. Within this design space, we directly examine 24 representative cases (Figure 5; Figure S3, Supporting Information), where we selected one ion with the highest conductance and the other eight cations. Figure 5 presents a set of highly diverse log(G) versus ΔV relationships for these cases, with exponential fits of the form $G=Ce^{\alpha \Delta V}$ shown as dashed lines.

Specifically, in the case of graphene membranes with 18‐crown‐6 pores with ${\mathrm{Na}}^{+}$ as the primary charge carrier, the addition of $K^{+}$, ${\mathrm{Rb}}^{+}$, ${\mathrm{Cs}}^{+}$, or ${\mathrm{NH}}_{4}^{+}$ induces voltage activated ${\mathrm{Na}}^{+}$ transport, while the addition of ${\mathrm{Ca}}^{2+}$ or ${\mathrm{Mg}}^{2+}$ results in voltage‐inactivated ${\mathrm{Na}}^{+}$ transport. Notably, α dramatically changes from –1.7 $V^{-1}$ (for ${\mathrm{Na}}^{+}$ alone) to 8.0 $V^{-1}$ upon the addition of 0.5 M ${\mathrm{NH}}_{4}^{+}$, and from ‐1.7 $V^{-1}$ to –7.1 $V^{-1}$ with 0.25 M ${\mathrm{Mg}}^{2+}$. Furthermore, the asymptotic conductance at near‐zero bias, C (in the exponential fit, $G=Ce^{\alpha \Delta V}$), decreases substantially from ∼400 pS to ∼1 pS in the presence of $K^{+}$, ${\mathrm{Rb}}^{+}$, or ${\mathrm{NH}}_{4}^{+}$. Not surprisingly, the functional role of each ion strongly depends on the pore type. For instance, the $B_{6}$ $N_{3}$ pore, which is only 0.1 Å larger than the 18‐crown‐6 pore, exhibits markedly different gating behavior such as voltage‐inactivated ${\mathrm{Rb}}^{+}$ transport in the presence of ${\mathrm{Mg}}^{2+}$ or ${\mathrm{Gdm}}^{+}$.

Each ion species can be categorized by its functional role within a given pore structure. In this work, we classified the ions into four broadly distinct functional roles: (1) primary charge transport carrier cations that permeate efficiently; (2) passive ions, which neither produce meaningful current nor influence the transport of the primary carriers; (3, 4) controller or “dopant” ions, which substantially influence the conduction of primary carriers without creating major current themselves. In this context, controller ions include those causing voltage activation (3) and inactivation (4). These functional roles are summarized in the color map shown in the upper‐right corner of Figure 5 for each considered pore structure, as determined based on how the conductance weights change with voltage and shown in Figure S3 (Supporting Information). We introduced the exponential relation for the conductance weight with respect to the voltage bias,

(5) $$w=Ae^{\beta \Delta V},$$

where the unit of β is $V^{-1}$. We defined tentative criteria based on the value of β: $\beta >1\;V^{-1}$ for voltage activation, $\beta <-1\;V^{-1}$ for voltage inactivation, and $-1\;V^{-1}<\beta <1\;V^{-1}$ for either the primary charge carrier or a passive ion, depending on whether it contributes substantially to the total current. Note that $\beta =1\;V^{-1}$ approximately results in a 10% increase in weight with a 100 mV applied voltage.

Targeted functional design of bicationic systems can be achieved by selecting appropriate ion–pore combinations. For example, to realize voltage‐activated ${\mathrm{Li}}^{+}$ transport, one would first identify a pore structure in which ${\mathrm{Li}}^{+}$ acts as the primary charge carrier—such as the 18‐crown‐6 pore or the $B_{10}$ $N_{6}$ vacancy—and then select a voltage‐activation controller within that pore, such as $K^{+}$ in the 18‐crown‐6 pore or ${\mathrm{Gdm}}^{+}$ in $B_{10}$ $N_{6}$ vacancy. As predicted, the 18‐crown‐6 pore system with a 1.0 M LiCl and 0.5 M KCl mixture exhibits a voltage‐activated ${\mathrm{Li}}^{+}$ transport behavior (see Figure S4, Supporting Information). Using this framework, we identify 15 potentially voltage‐activated systems, including voltage‐activated ${\mathrm{Li}}^{+}$, ${\mathrm{Na}}^{+}$, and $K^{+}$ channels. Similarly, 36 systems potentially exhibiting voltage‐inactivation are predicted, including voltage‐inactivated ${\mathrm{Li}}^{+}$, ${\mathrm{Na}}^{+}$, $K^{+}$, ${\mathrm{Rb}}^{+}$, ${\mathrm{Cs}}^{+}$, and ${\mathrm{NH}}_{4}^{+}$ channels. This role‐based classification can reduce the combinatorial design space, enabling the rational development of liquid‐state ionic devices with tailored functionalities.

While this study focuses on binary cationic systems represented by individual devices under symmetric electrolyte conditions, the design space can be further broadened by incorporating adjusting the concentration of dopant (controller) ion (see Figure S5), asymmetric ion concentrations, ternary and higher‐order mixtures (see the more complex behavior observed in tertiary mixtures in Figure S6), as well as circuit‐level architectures such as serial or parallel arrangements. Additionally, non‐ionic species can modulate ion transport by selectively interacting with pores [39], analogous to neurotransmitters regulating ion channels in biological systems. Considering that subtle ion‐pore interactions play a crucial role in doping, advanced computational simulations that can accurately capture these interactions, such as those incorporating polarization effects, may be necessary for quantitative assessment. This vast design space of liquid‐state ionics, achievable through liquid‐state “doping” offers a wide spectrum of functional diversity that may easily surpass that of solid‐state electronics. Together, these possibilities define a wide and largely unexplored landscape for advancing the engineering of liquid‐state ionic devices.

For future experimental validation, it is critical to understand that implementing these multi‐ion functionalities requires precise fabrication of porous membranes that enable barrier‐limited transport for at least one of the ionic species present in the solution. The corresponding pore architectures should provide well‐defined binding sites for the “dopant” ions while permitting efficient permeation of the primary transport carriers. The model structures presented in this work have already been successfully synthesized—for example, graphene‐embedded crown ether [40] and monolayer hBN [41, 42]. A remaining challenge is the fabrication of membranes with uniform pore arrays. Alternatively, because pore structure uniformity is essential, porous materials such as MOFs, COFs, and $C_{2}N$ membranes—featuring intrinsically crystalline porous structures, may be particularly promising. Membrane thickness is another key consideration: overly thick membranes with strong binding affinity may produce negligible currents or demand unreasonably large voltages to release trapped ions. Although atomically thin membranes (one to three layers) appear to be generally suitable, atomic thinness may not be a stringent requirement.

## 3 Conclusion

The brain leverages a rich diversity of ions and molecules for sophisticated neural signaling—a capability largely untapped in synthetic devices. Our work demonstrates that this electrochemical diversity, often a minor factor in diffusion‐limited systems, becomes a powerful design parameter governing ion transport in the barrier‐limited transport regime. By systematically investigating 33 single‐salt and 24 binary‐salt systems using all‐atom molecular dynamics, we show that competitive ion transport across Å‐pores gives rise to voltage activation, voltage inactivation, pulse generation, and synaptic‐like potentiation. This liquid‐state doping within the barrier‐limited transport regime creates a vast design space that scales exponentially with the number of ion species, as $2^{N_{\text{ion}}}$ per pore structure. Our findings underscore the unexplored potential of liquid‐state ionic devices enabled by electrochemical diversity, highlighting their promise for emulating the functional diversity of biological ion transport. Looking further ahead, the exponential scaling of functional diversity with ionic composition may promise a fundamental advantage of ion‐based systems over their purely electronic counterparts. Accordingly, our results support the concept of ion‐based computing platforms as a highly effective route toward advanced, scalable neuromorphic devices. Furthermore, because biological neural signaling inherently operates through multi‐ion, barrier‐limited transport, our results suggest that similar mechanistic principles may underlie information processing in living systems. This connection invites new lines of inquiry spanning neuromorphic engineering, neuroscience, and molecular biology.

## Conflicts Of Interest

The authors declare no conflict of interest.

## Supporting information


**Supporting Information** smll72492‐sup‐0001‐SuppMat.pdf.

## Data Availability

The data that support the findings of this study are available from the corresponding author upon reasonable request.
