# Supplementary material for: Leveraging Electrochemical Diversity in Engineering Liquid‐State Ionic Devices for Neuromorphic Computing
Source: Small. 2026 Jan 21;22(16):e11663. doi: 10.1002/smll.202511663 (PMC12994564; doi:10.1002/smll.202511663)
Supplement: Supplementary file 1 — Supporting Information smll72492‐sup‐0001‐SuppMat.pdf. [file SMLL-22-e11663-s001.pdf]

Supporting Information: Leveraging Electrochemical Diversity in Engineering Liquid-State Ionic Devices for Neuromorphic Computing

Yechan Noh*,†,‡,¶ and Alex Smolyanitsky†

†*Applied Chemicals and Materials Division, National Institute of Standards and Technology, Boulder, CO 80305, USA*

‡*Department of Physics, University of Colorado Boulder, Boulder, CO 80309, USA*

¶*Department of Aerospace and Mechanical Engineering, University of Notre Dame, Notre Dame, IN 46556, USA* [a]

E-mail: ynoh@nd.edu

[a]Current affiliations: National Institute of Standards and Technology and University of Notre Dame

S1 Molecular Dynamics Simulations

All-atom molecular dynamics simulations were performed using the GPU-accelerated GROMACS software package (version 2024.4).[1,2] The graphene sheet functionalized with 18-crown-6-like pores was modeled using previously established parameters,[3,4] with partial charges assigned to oxygen (-0.4e) and neighboring carbon (+0.2e) atoms to ensure a net neutral pore structure. Nine equally spaced 18-crown-6 pores were embedded in a graphene sheet with dimensions 6.39 nm by 5.903 nm in the x- and y-directions. The height of the rectangular simulation cell was 6.0 nm. The hBN membrane was modeled using parameters from Rajan et al.,[5] with dimensions of 5.974 nm by 6.036 nm by 6.0 nm. For hBN membranes with vacancy arrays, partial charges were assigned to bulk nitrogen (-0.9e) and boron (+0.9e) atoms, while edge nitrogen atoms carried a reduced charge (-0.6e), resulting in a zero total charge of the porous region. Simulations were conducted with periodic boundary conditions applied in XYZ. All membranes were position-restrained along the perimeter to prevent drifting during simulation. Water molecules were modeled using the TIP4P model.[6] All non-bonded interactions were treated using the CHARMM36 force field.[7,8] The guanidinium ion (Gdm^+) was modeled using parameters from Camilloni et al.[9] Electrostatic interactions were handled using the particle-particle–particle-mesh scheme with a short-range cut-off radius of 1.2 nm; the same cut-off radius was used to resolve the Lennard-Jones interactions. The initial system was energy-minimized using the steepest descent algorithm, followed by a 2 ns NPT equilibration at 300 K and 1 bar. The Berendsen barostat and velocity-rescaling thermostat were used to control pressure and temperature, respectively. Ion transport simulations were conducted in the NVT ensemble under a uniform electric field applied in the z-direction. For alternating current (AC) bias, a sinusoidal electric field $E(t) = E_0 cos(\omega t)$ was applied following a 10 ns pre-relaxation under a constant field E_0. Ion flux and current were calculated using the methods described in the supporting information of previous work.[10] Molecular visualization was performed using OVITO.[11]

S2 Discussion on the barrier formation in a single-salt system

The formation of transport barrier of ions involves multiple factors, including the interactions between the ions and surrounding water molecules, ion-pore interactions, entropic energy changes during transport, and even water-water and pore-water interactions.[12] For the systems we present, one of the major contributors to the transport barrier is dehydration. For example, in the case of B_6N_3 vacancy (Fig. 2c, g in the main text), the more robust hydration shells of smaller monovalent ions result in considerably lower conductance compared to their larger counterparts ($G_{\mathrm{Li}^+} < G_{\mathrm{Na}^+} < G_{\mathrm{K}^+} \approx G_{\mathrm{Rb}^+} \approx G_{\mathrm{Cs}^+}$). The relative stability of hydration and the size of ion hydration shells can be assessed from the height and position of the first peak in the radial distribution functions, as shown in Fig. S1. Note that divalent cations exhibit more complex transport behavior due to their highly stable hydration shells (for comparison, the hydration enthalpy of K^+ and Mg^{2+} is -322 kJ/mol and -1921 kJ/mol, respectively[13]). As a result, divalent ions typically permeate as fully hydrated complexes under realistic driving forces, and their passage through narrow pores, such as $B_{10}N_6$ vacancies in hBN, restricts the possible configurations of hydration waters, creating a substantial entropic energy barrier near the pore.[12]

To directly access the transport barrier associated with ion transport, we conducted extensive molecular dynamics simulations using umbrella sampling to obtain the potential of mean force (PMF) profiles of each ion-pore pair, as shown in Fig. S2. Note that the transport rate is exponentially sensitive to the rate-setting energy barrier, as described in the main text (discussions around Equation 2); a change of $\sim 2.3K_BT$ in PMF can result in an order of magnitude change in ion current. The PMF itself should contain the essence of ion transport and trapping behavior; however, a theoretical framework is lacking to accurately predict the rate of ion transport and trapping time (or occupancy) with only PMF. Therefore, current best practice is to use both PMF and trajectory analysis of transport simulations

to gain a comprehensive understanding of the molecular mechanisms and properties (e.g., trapping time). Thus, the simplified PMF data shown in Fig. 2 in the main text are a result of both analyses.

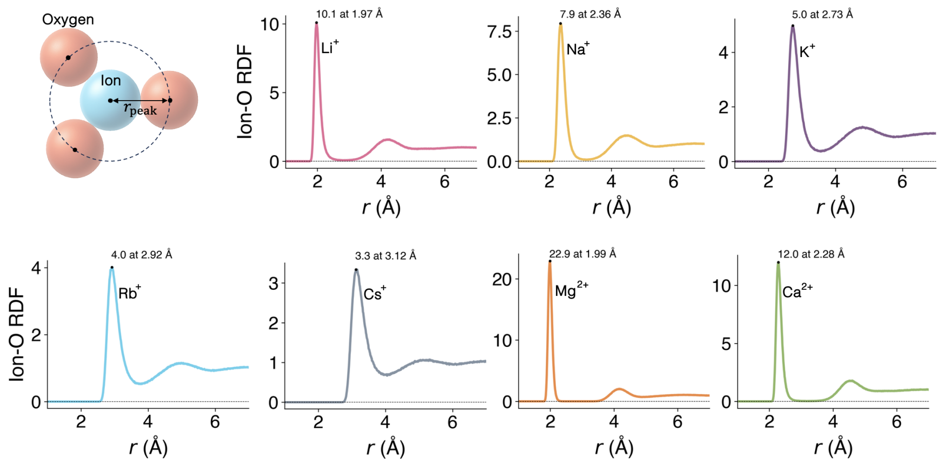

Figure S1: **Ion–oxygen radial distribution functions in water.** The schematic (top left) illustrates the first RDF peak location r_{peak}, defined as the statistically significant separation between the cation and the water oxygens in the first shell. Panels show ion–oxygen RDFs for eight cations: (**top row**) Li^+, Na^+, K^+, and (**bottom row**) Rb^+, Cs^+, Mg^{2+}, and Ca^{2+}. Each plot displays the RDF peak value and corresponding radial separation.

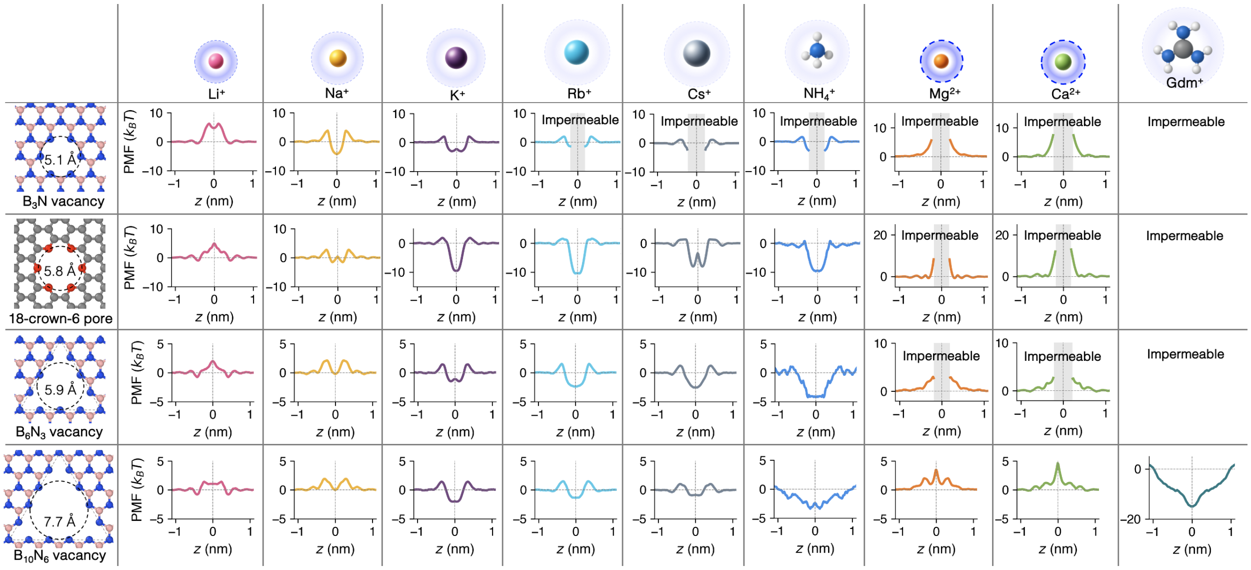

Figure S2: **Potential of mean force profiles for various cations across four Å-scale pores.** Rows correspond to pore structures, for which PMFs are calculated, including a B_3N vacancy, an 18-crown-6 pore, a B_6N_3 vacancy, and a $B_{10}N_6$ vacancy, as illustrated in the leftmost column. Columns display the PMF profiles of individual ions along the pore axis. Gray shaded regions indicate ion-inaccessible zones where sufficient sampling could not be performed. "Impermeable" denotes cases in which ion translocation was not observed during ion transport simulations. PMFs were computed using umbrella sampling at 300 K for systems containing a single cation and either one or two anions, depending on the cation's valency.

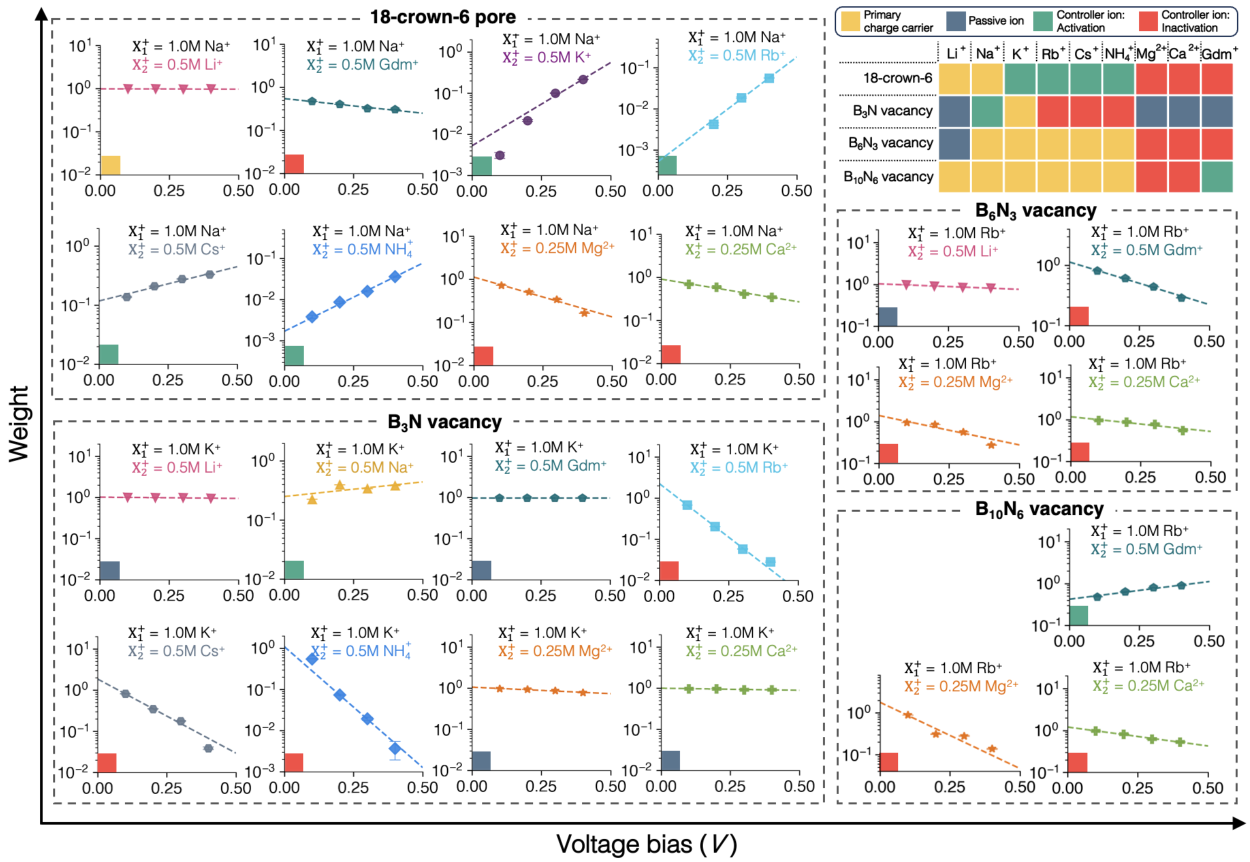

Figure S3: **Voltage-dependent conductance weights for diverse cation pairs in four distinct Å-scale pores.** Each panel shows the relationship between $\log(w)$ and ΔV. The four pore types are: 18-crown-6 pore (top left), B_3N vacancy (bottom left), B_6N_3vacancy (middleright), and $B_{10}N_6$ vacancy (bottom right). Dashed lines represent exponential fits of the form $w = Ae^{\beta \Delta V}$, where the unit of β is V^{-1}. The colored square in the bottom left corner of each plot indicates the functional role of the secondary ion (X_2^+) shown in the top right color map.

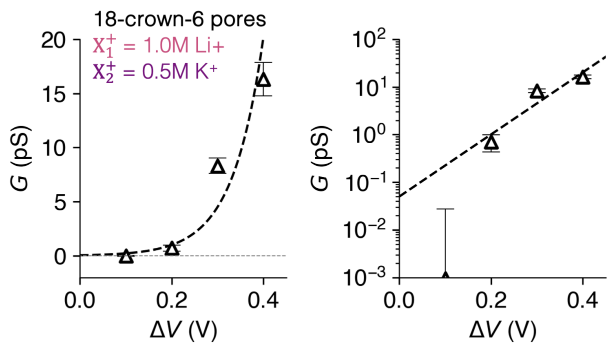

Figure S4: **Voltage-activated Li^+ transport in 18-crown-6 pores in a 1.0 M LiCl and 0.5 M KCl mixture** Left: Conductance–voltage bias curve (linear scale). Right: Conductance–voltage bias curve (semi-logarithmic scale).

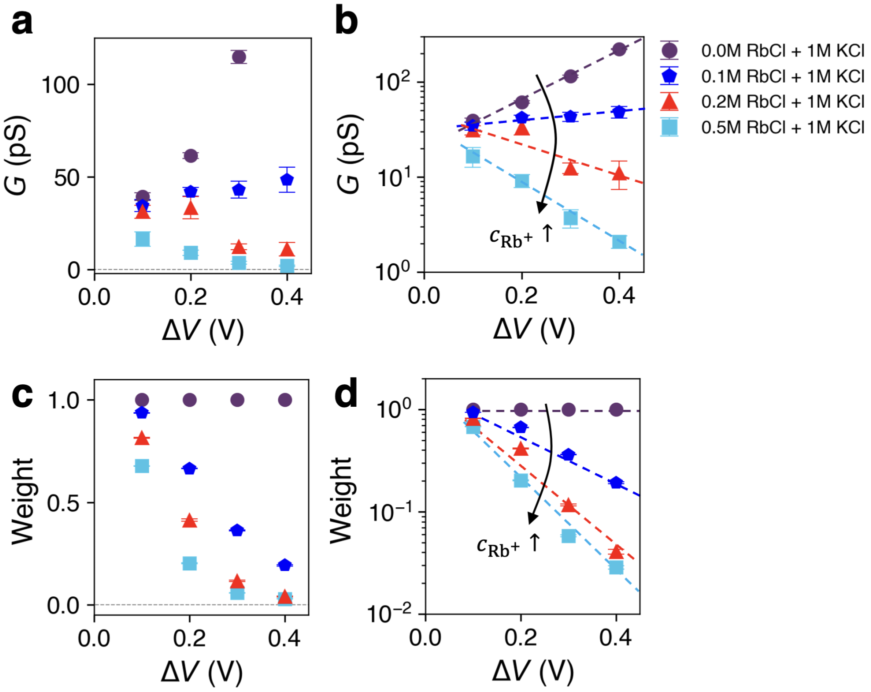

Figure S5: **Effect of Rb^+ concentration on voltage inactivation in a B_3N vacancy system with mixed KCl and RbCl electrolytes.** (**a**, **b**) Conductance-voltage (G-ΔV) characteristics at varying Rb^+ concentrations (0 to 0.5 M) in 1 M KCl. (**a**) Linear scale and (**b**) semi-logarithmic scale. (**c**, **d**) Weight-voltage (w-ΔV) curves. (**c**) Linear scale and (**d**) semi-logarithmic scale.

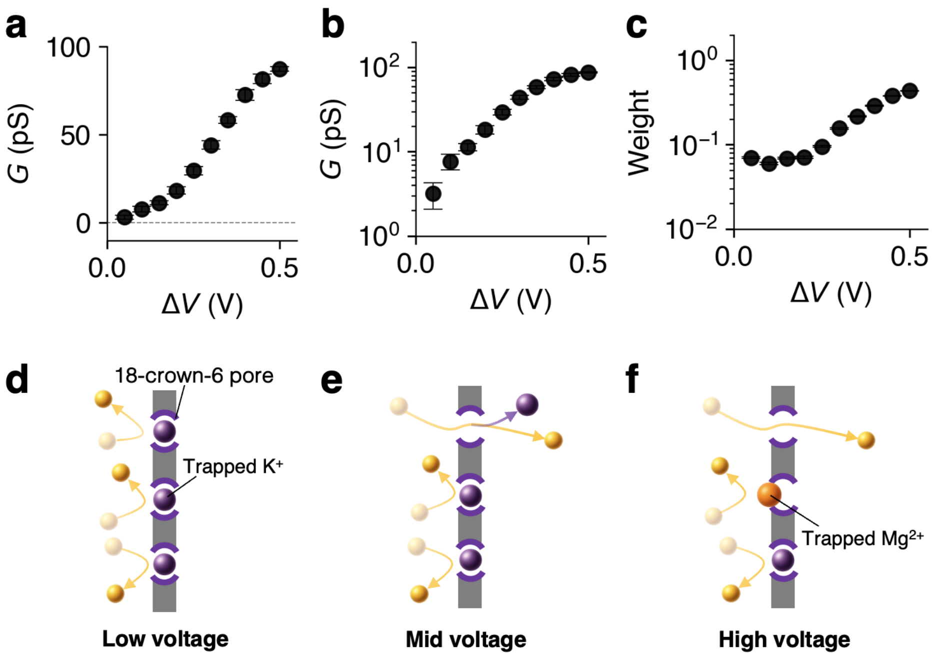

Figure S6: **Ion transport behavior involving three cations (1.0M NaCl, 0.5M KCl, and 0.25M $MgCl_2$) in 18-crown-6 pore system.** (**a**–**b**) Ion conductance as a function of voltage bias plotted using a linear (**a**), and (**b**) logarithmic scale ($\log(G)$–ΔV). (**c**) Voltage dependence of normalized conductance weight ($\log(w)$–ΔV). (**d**–**f**) Schematics of tricationic transport and cation binding across three voltage regimes: low voltage (**d**), mid voltage (**e**), and high voltage (**f**) .

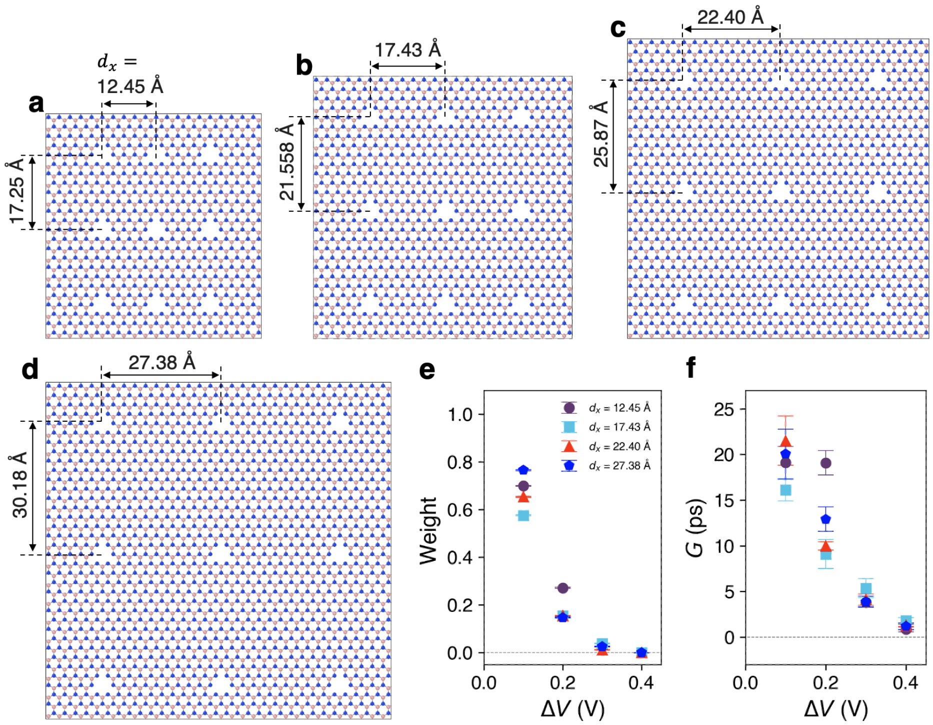

Figure S7: **Effect of pore spacing on voltage-inactivated gating behavior in B3N vacancy arrays** (**a-d**) Atomistic configurations of hBN membranes containing periodic B_3N vacancy arrays with varying pore spacing: d_x= 12.45 Å, 17.43 Å, 22.40 Å, and 27.38 Å, respectively. (**e**) Conductance weight as a function of applied bias for each pore spacing. (**f**) CCorresponding single-pore conductance (averaged over nine pores) as a function of applied bias.

References

(1) Abraham, M. J.; Murtola, T.; Schulz, R.; Páll, S.; Smith, J. C.; Hess, B.; Lindahl, E. GROMACS: High performance molecular simulations through multi-level parallelism from laptops to supercomputers. *SoftwareX* **2015**, *1*, 19–25.

(2) Páll, S.; Zhmurov, A.; Bauer, P.; Abraham, M.; Lundborg, M.; Gray, A.; Hess, B.; Lindahl, E. Heterogeneous parallelization and acceleration of molecular dynamics simulations in GROMACS. *The Journal of Chemical Physics* **2020**, *153*.

(3) Smolyanitsky, A.; Paulechka, E.; Kroenlein, K. Aqueous Ion Trapping and Transport in Graphene-Embedded 18-Crown-6 Ether Pores. *ACS Nano* **2018**, *12*, 6677–6684.

(4) Fang, A.; Kroenlein, K.; Riccardi, D.; Smolyanitsky, A. Highly mechanosensitive ion channels from graphene-embedded crown ethers. *Nature Materials* **2019**, *18*, 76–81.

(5) Govind Rajan, A.; Strano, M. S.; Blankschtein, D. Ab initio molecular dynamics and lattice dynamics-based force field for modeling hexagonal boron nitride in mechanical and interfacial applications. *The Journal of Physical Chemistry Letters* **2018**, *9*, 1584–1591.

(6) Jorgensen, W. L.; Chandrasekhar, J.; Madura, J. D.; Impey, R. W.; Klein, M. L. Comparison of simple potential functions for simulating liquid water. *The Journal of Chemical Physics* **1983**, *79*, 926–935.

(7) Huang, J.; Rauscher, S.; Nawrocki, G.; Ran, T.; Feig, M.; De Groot, B. L.; Grubmüller, H.; MacKerell Jr, A. D. CHARMM36m: an improved force field for folded and intrinsically disordered proteins. *Nature methods* **2017**, *14*, 71–73.

(8) MacKerell Jr, A. D.; Bashford, D.; Bellott, M.; Dunbrack Jr, R. L.; Evanseck, J. D.; Field, M. J.; Fischer, S.; Gao, J.; Guo, H.; Ha, S.; others All-atom empirical potential for

molecular modeling and dynamics studies of proteins. *The journal of physical chemistry B* **1998**, *102*, 3586–3616.

(9) Camilloni, C.; Rocco, A. G.; Eberini, I.; Gianazza, E.; Broglia, R.; Tiana, G. Urea and guanidinium chloride denature protein L in different ways in molecular dynamics simulations. *Biophysical journal* **2008**, *94*, 4654–4661.

(10) Noh, Y.; Smolyanitsky, A. Memristive Response and Capacitive Spiking in Aqueous Ion Transport through Two-Dimensional Nanopore Arrays. *The Journal of Physical Chemistry Letters* **2024**, *15*, 665–670.

(11) Stukowski, A. Visualization and analysis of atomistic simulation data with OVITO–the Open Visualization Tool. *Modelling and Simulation in Materials Science and Engineering* **2009**, *18*, 015012.

(12) Noh, Y.; Riccardi, D.; Smolyanitsky, A. Entropic modulation of divalent cation transport through porous two-dimensional materials. *arXiv preprint arXiv:2504.05569* **2025**,

(13) Smith, D. W. Ionic hydration enthalpies. *Journal of Chemical Education* **1977**, *54*, 540.
